# Medical Record Survey after Comprehensive Health Checkup Referral and Its Contribution to the Early Detection of Cancer

**DOI:** 10.3390/jpm14010059

**Published:** 2023-12-30

**Authors:** Yoko Yamanouchi, Takaaki Senbonmatsu, Takumi Yamaguchi, Ikuo Inoue, Seiichi Goto, Tomoyuki Soma, Yoshiaki Maruyama, Masaki Adachi, Nozomi Shinozuka, Toshihiro Muramatsu

**Affiliations:** 1 Preventive Medicine Center, Saitama Medical University Hospital, Saitama 350-0495, Japantsoma@saitama-med.ac.jp (T.S.);; 2 Department of Rehabilitation Medicine, Saitama Medical University School of Medicine, Saitama 350-0495, Japan; 3 Research Administration Center, Saitama Medical University, Saitama 350-0495, Japan; senbont@saitama-med.ac.jp (T.S.);; 4 Department of Endocrinology and Diabetology, Saitama Medical University School of Medicine, Saitama 350-0495, Japan; 5 Department of Respiratory Medicine, Saitama Medical University School of Medicine, Saitama 350-0495, Japan; 6 Unicus-Kawagoe Preventive Medicine Center, Saitama 350-0495, Japan; 7 Department of General Surgery, Saitama Medical University School of Medicine, Saitama 350-0495, Japan

**Keywords:** comprehensive health checkup, medical record survey, cancer screening, COVID-19, abdominal ultrasound

## Abstract

Comprehensive health checkups in Japan are a preventive method to detect cancer and metabolic diseases. Unlike group medical examinations, individual examinations in health checkups are possible, with additional tests possible for disease detection. However, it is difficult to accurately ascertain the results from only the report after referral to a medical institution in individuals suspected of having cancer who need to be examined. We aimed to conduct a medical record survey of patients referred to the Hospital after undergoing a comprehensive health checkup and investigate the contribution of comprehensive health checkups to the detection of cancer more accurately. The subjects were 1763 examinees who were referred to various departments of our hospital because of doubtful cancer from 23,128 examinees who underwent comprehensive health checkups in our center from January 2018 to December 2022 for 5 years. The medical record survey demonstrated that cancer was detected in more than twice as many individuals as reported and other sources. Early-stage cancers require a significantly longer time to establish a definitive diagnosis. In conclusion, short-term reports from the referring hospital are insufficient for a final diagnosis, and long-term follow-up is extremely important to increase the diagnosis rates of cancer for comprehensive health checkups.

## 1. Introduction

Medical services in Japan are remarkably different from those worldwide. In Japan, a universal health insurance system has been adopted, in which all citizens are covered by public health insurance and can freely select their medical institutions [1]. Japanese citizens are required to enroll in health insurance, and workers are required to undergo periodic health examinations once a year in accordance with the Occupational Health and Safety Law [2]. Specific health examinations have also been conducted in individuals aged 40–74 years for the prevention and early detection of lifestyle-related diseases [3]. However, these checkups have a limited number of items. In addition, cancer screening is promoted by the government as part of its policy, but its screening rate is low [4].

In Japan, the comprehensive health checkup (in Japanese, “Ningen Dock”, the comprehensive health checkup) is a special system for early detection of diseases, conducted separately from these standardized universal health checkups, and can be taken on a voluntary basis for a fee. It is conducted at 1727 facilities throughout Japan, with approximately 3.7 million individuals participating annually [5]. The main objectives of the comprehensive health checkup system are to maintain good health via early detection of cancer, detection of metabolic diseases, and checking the overall health status [5]. This aims to detect diseases that cannot be detected using general medical examinations alone. Therefore, comprehensive health checkups include cancer screening.

The comprehensive health checkup at the Preventive Medicine Center (the Center) of the Saitama Medical University Hospital (the Hospital) also aims to detect cancer and many other diseases via several tests.

Basic laboratory tests in the comprehensive health checkup at the Center include the following medical and physical examinations: visual acuity, intraocular pressure, fundus, hearing, blood pressure, electrocardiogram (ECG), pulmonary function, and blood sampling (glucose, lipids, renal function, uric acid, electrolytes, liver function, hepatitis B virus, general blood, C-reactive protein) evaluations, abdominal ultrasonography, chest radiography, gastric radiography, urinalysis and fecal occult blood test.

In addition, additional test items that can be freely selected include the following tests: gastrointestinal endoscopy, head computed tomography (CT), thoracoabdominal CT, sputum cytology, hepatitis C virus assessment, ABC screening (*Helicobacter pylori*), stress electrocardiogram, echocardiography, brain natriuretic peptide (NT-Pro) evaluation, arteriosclerosis set assessment, vascular age test, sleep apnea test, three-dimensional fundus examination, bone density assessment, locomotive health check evaluation, positron emission tomography (PET) scan, prostate-specific antigen (PSA) assessment, comprehensive gynecological examination, and breast screening. The accuracy of these measurements is assured by the Laboratory Quality Control Survey conducted by the Japan Society of Health Examination and Promotion.

A characteristic feature of comprehensive health checkups at university hospitals is that several specialists cooperate with each other, and when an abnormality is found, it can be referred directly to the respective departments, taking advantage of the merits of a general hospital. In the Center, examinees with abnormal findings are often referred directly to hospital departments. Although we would have received a prompt response after referral, in several cases, the final results were unknown because the examination had not been conducted at the time the report was prepared, and the patient was told that a closer examination would be conducted in the future. Prior to the medical record survey, the number of cancer cases was ascertained using reports from referring medical institutions. In some cases, the number of cancer cases was ascertained using return questionnaires or phone calls from the patients themselves. We have reported the number of cancer cases detected annually to the Japan Society of Ningen Dock and other organizations; however, this has not been an accurate figure [6]. To obtain an accurate number of cancer cases, a medical record survey was conducted on patients who were referred to the hospital. The Center’s contribution to the early detection of cancer was also discussed.

The coronavirus disease 2019 (COVID-19) pandemic also has a significant effect on Japan and other countries. The Hospital was also severely affected, and comprehensive health checkups were suspended for 2 months in April and May 2020, when a state of emergency was declared in Japan [7]. The comprehensive health checkup was reopened in June; however, infection control measures and limitations on the number of examinees continue. The effect of the COVID-19 pandemic on the number of examinations and cancer detection rates during comprehensive health checkups was also examined.

It is difficult to accurately ascertain the results from only the report after referral to a medical institution in individuals suspected of having cancer who need to be examined. This is because some people do not know the details of the results even if they have been examined. We hypothesized that more cancers may have been detected than have been reported. The aim of the present study was to conduct a medical record survey of patients referred to the Hospital after undergoing a comprehensive health checkup and investigate the contribution of comprehensive health checkups to the detection of cancer more accurately. This is important to objectively evaluate the significance of comprehensive health checkups and to help people understand it.

## 2. Materials and Methods

### 2.1. Ethics Statements

This study design was approved by the Ethical Review Committee of Saitama Medical University Hospital (approval number: 2022-097). As this was a retrospective study, the requirement for informed consent was waived; however, information regarding the study was made public, and the participants were guaranteed the opportunity to refuse participation.

### 2.2. Participants

This study comprised 1763 patients (mean age, 62.0 years; 938 men and 825 women) referred to various departments of the Hospital for suspected cancer among 23,128 examinees who underwent physical examinations at the Center over a 5-year period from January 2018 to December 2022.

### 2.3. Methods

Medical Record Survey

A medical record survey was conducted according to the following steps.

Step 1: Check if the patient has cancer.

After checking all patients, the number of patients referred to the Hospital (A), the number of cancers detected after referral (B), and the cancer detection rate (B × 100/A) were calculated.

Step 2: For patients who had cancer, check the following items:

1. Patients whose results were originally known from reports or personal reports (group before the survey) or patients whose results were identified by a medical record survey (group after the survey);
2. Sex;
3. Age;
4. Cancer type;
5. Cancer progression: Early stage: Stage 0–1 or equivalent; Advanced stage: Stage 2 or higher, or equivalent; or Unknown: Unknown only in the medical records of the hospital;
6. Time to confirmed cancer diagnosis: Time from the date of the comprehensive health checkup to confirmed cancer diagnosis (in months);
7. Reason for referral: The Center’s laboratory tests that were the reason for the referral;
8. Progress: Gather information on progress to diagnosis.

Step 3: For the referred patients (group after the survey) who were found to have cancer after the medical record survey, the causes that could not be ascertained before the survey were classified into the following types:

9. Results not reported;
10. Inspection not performed at the time the report was prepared because of the time it takes to complete the inspection;
11. If the type of cancer (malignant or benign) was unclear, cancer found after follow-up
12. Referred to another hospital and found to have cancer;
13. The initial biopsy was benign but later proved to be malignant;
14. Others.

Inclusion criteria:

- Include a new flare-up of a previous cancer.

Exclusion criteria:

- Exclude cancers that are different from those for which the referral was intended, when they are detected by chance examination.
- Cancers undergoing treatment are also excluded.

We also sought to determine whether there were any changes in cancer detection rates before and after the COVID-19 pandemic. The examinees of the comprehensive health checkup at the Center, number of patients referred to the Hospital (A), number of cancers detected after referral (B), and cancer detection rate (B × 100/A) were tabulated for each year from 2018 to 2022.

### 2.4. Statistical Analyses

Variables are expressed as means ± standard deviations. Differences between the two groups were compared using a *t*-test and chi-square test. For correlations between factors, Pearson’s correlation coefficient (r) was calculated for correlations between parametric variables, and Spearman’s correlation coefficient (ρ) was calculated when nonparametric variables were present. Correlation coefficients (r) and (ρ) were considered to be correlated when they were ≥0.2. A multivariate binomial logistic regression analysis was performed, with ‘Cancer Patients’ and ‘Early-Stage Cancer’ as the dependent variables. The independent variables included in the multivariate model were ‘Year’, ‘Sex’, ‘Age’, and ‘Survey’ for the ‘Cancer Patients’ model, and ‘Year’, ‘Age’, ‘Sex’, and ‘Period’ for the ‘Early- Stage Cancer’ model, respectively. The forced entry method was used for model selection. Coefficients, odds ratios (ORs), 95% confidence intervals (CIs), and *p* values were calculated. The Hosmer–Lemeshow test yielded values of 0.415 and 0.156, respectively.

Statistical Package for the Social Sciences version 24 (IBM Corp., Armonk, NY, USA) was used for all the statistical analyses. Sex was considered a binary dummy variable (1/2), with males assigned a status of 1 and females assigned a status of 2. Whether the patient had cancer was considered a binary dummy variable (0/1), with 1 assigned to the status of patients with cancer and 0 to all others. Whether the patient was found before or after the medical reports survey was a dichotomous dummy variable (1/2), with 1 assigned to status before the medical reports survey and 2 assigned to status after the medical reports survey.

For year-to-year changes, a one-way analysis of variance was used to identify 5-year differences.

## 3. Results

### 3.1. Identification of Patients with Cancer According to the Survey: Age, Sex, and Relationship before and after the Survey

During the 5-year period from January 2018 to December 2022, of the 23,128 examinees who underwent comprehensive health checkups at the Center, 3081 were referred to hospitals, including the Hospital, due to findings such as suspected cancer. Of these patients, 1763 (mean age, 62.0 ± 11.7 years; 938 men and 825 women) were referred to various departments of the Hospital.

The mean age of all examinees was 62.0 ± 11.7 years. The mean ages for males and females were 64.3 ± 11.5 and 59.4 ± 11.4, respectively, with a slightly higher mean age for males than for females (*p* < 0.001).

Of the 1763 referred patients, 33 were originally aware of the results via reports or personal reports (group before the survey), and 56 patients were identified via medical record surveys (group after the survey). The Center’s referrals led to the discovery of 89 patients with cancer (mean age, 67.2 ± 8.8 years; 61 men and 28 women). The cancer detection rate increased from 1.87% before the survey to 5.05% after.

Basic information about 1763 patients referred to the Hospital is presented in Table 1.

The mean age of the 89 patients with cancer was 67.2 ± 8.8 years, which was significantly higher than that of the patients without cancer (61.7 ± 11.8 years) (*p* < 0.001).

In total, 61 of the 938 males and 28 of the 825 females had cancer, with significant sex differences (*p* = 0.002). The cancer detection rate was significantly higher in men than in women (6.96% vs. 3.39%) (*p* = 0.002).

A comparison of the variables between the patients with and without cancer is presented in Table 2.

A multivariate binomial logistic regression analysis was performed to determine whether there was an effect on patients with cancer, sex, age, and year of examination, and it was found that being male (OR = 1.626, *p* = 0.042) and older (OR = 1.043, *p* < 0.001) were independently and significantly associated. There were no differences according to the year of inspection.

The relationship to the presence or absence of cancer is shown in Table 3.

The mean age of the 33 patients with cancer before this study was 66.6 ± 9.2 years, and the mean age of the 56 patients with cancer after this study was 67.7 ± 8.7 years, with no significant difference between them. The average time to confirm cancer diagnosis was 2.76 ± 2.45 months before the survey, but it was significantly longer after the survey at 4.80 ± 4.88 months (*p* = 0.010).

A comparison of the variables between patients with cancer before and after the survey is shown in Table 4.

### 3.2. Cancer Type

The numbers of patients after the survey (number before the survey) were 10 (2) for lung cancer, 2 (1) for esophageal cancer, 12 (7) for stomach cancer, 18 (9) for colorectal cancer, 3 (1) for pancreatic cancer, 6 (3) for renal cancer, 6 (2) for bladder cancer, 13 (3) for prostate cancer, 9 (5) for breast cancer, 6 (0) for hematological malignancy (4(0) for malignant lymphoma, 1 (0) for malignant myeloma, 1 (0) for leukemia), and 4 (0) for others [2 (0) for sarcoma, 2 (0) for others], with a total number of 89 (33) patients.

**Figure 1:**
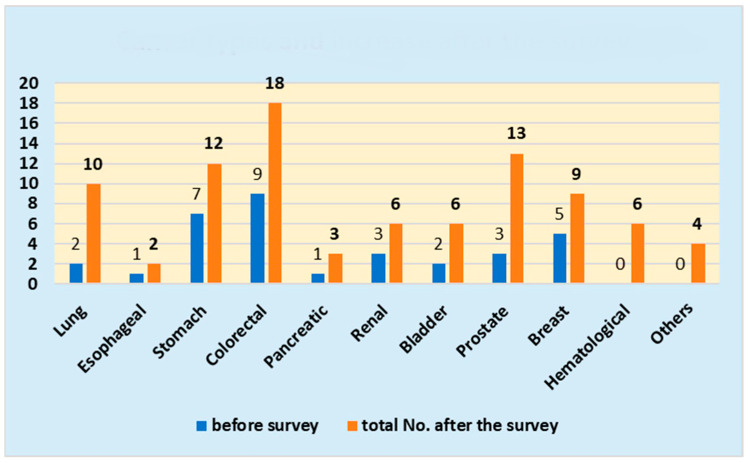
Cancer types and increase in incidence after the survey.

The cancer types and increase after the survey are shown in Figure 1. 

### 3.3. Cancer Stages

Of the 89 patients, 50 (56.2%) had early-stage cancer, 25 (28.1%) had advanced cancer, and 14 (15.7%) had an unknown stage.

Before the survey, 17 patients had early-stage cancer, 13 had advanced disease, and 3 had an unknown stage.

After the survey, an additional 33 patients with early-stage cancer, 12 with advanced cancer, and 11 with unknown stage were identified. Some patients were referred to other medical facilities by the Hospital; therefore, their disease stages could not be confirmed. Those identified after the survey tended to have a higher percentage of early-stage cancers; however, this was not statistically significant.

The mean age of the 50 patients with early-stage cancer was 66.5 ± 9.3, and the mean age of the 25 patients with advanced cancer was 69.4 ± 6.4, with no significant difference between them (*p* = 0.090). For early-stage (*n* = 50) and advanced (*n* = 25) cancers, the mean times to confirmed cancer diagnosis were 4.98 ± 5.12 months and 2.28 ± 1.67 months, respectively. When a *t*-test was performed to determine whether there was a difference between the two groups, early-stage cancers took longer to diagnose (*p* = 0.001).

A comparison of the variables between patients with early-stage cancer and those with advanced cancer is shown in Table 5.

The average times to confirmed cancer diagnosis were 3.59 ± 3.14 months and 5.70 ± 5.80 months for pre-survey (*n* = 17) and post-survey (*n* = 33) early-stage cancers, respectively. There was a trend toward a longer post-survey period, but the difference was not significant (*p* = 0.102).

A multivariate binomial logistic regression analysis for early-stage cancer showed a significantly associated only in the time to confirmed cancer diagnosis (OR = 1.228, *p* = 0.037). There was no significant other than time to confirm cancer diagnosis.

The correlations between risk factors and early-stage cancer are shown in Table 6.

Whether the patient was found before or after the medical reports survey was a dichotomous dummy variable (1/2), with 1 assigned to status before the medical reports survey and 2 assigned after the medical reports survey.

### 3.4. Reasons for Referral

The reasons for referral of 1763 patients (89 patients with cancer) were as follows: fecal occult blood, 521 (19) (cancer detection rate, 3.65%); abdominal ultrasonography, 272 (21) (cancer detection rate, 7.72%); gastric ultrasonography, 147 (4) (cancer detection rate, 2.72%); gastric endoscopy, 69 (7) (cancer detection rate, 10.14%); chest ultrasonography, 161 (8) (cancer detection rate, 4.97%); chest CT, 109 (5) (cancer detection rate, 4.59%); internal medicine consultation, 39 (0) (cancer detection rate, 0%); brain CT, 2 (0) (cancer detection rate, 0%); chest CT, 15 (0) (cancer detection rate, 0%); internal medicine consultation, 39 (0) (cancer detection rate, 0%); brain CT, 2 (0) (cancer detection rate, 0%); abdominal CT, 15 (0) (cancer detection rate, 0%); brain CT, 2 (0) (cancer detection rate, 0%); medical examination, 39 (0) (cancer detection rate, 0%); general blood assessment, 61 (2) (cancer detection rate, 3.28%); liver function evaluation, 17 (0) (cancer detection rate, 0%); hepatitis virus assessment, 6 (0) (cancer detection rate, 0%); *H. pylori* test, 17 (0) (cancer detection rate, 0%); renal function assessment, 30 (1) (cancer detection rate, 3.33); urinalysis, 33 (2) (cancer detection rate, 6.06%); pulmonary function assessment, 31 (0) (cancer detection rate, 0%); sputum cytology evaluation, 1 (0) (cancer detection rate, 0%); PSA evaluation, 52 (11) (cancer detection rate, 21.15%); breast screening, 49 (9) (cancer detection rate, 18.37%); and gynecological examination, 131 (0) (cancer detection rate, 0%).

**Figure 2:**
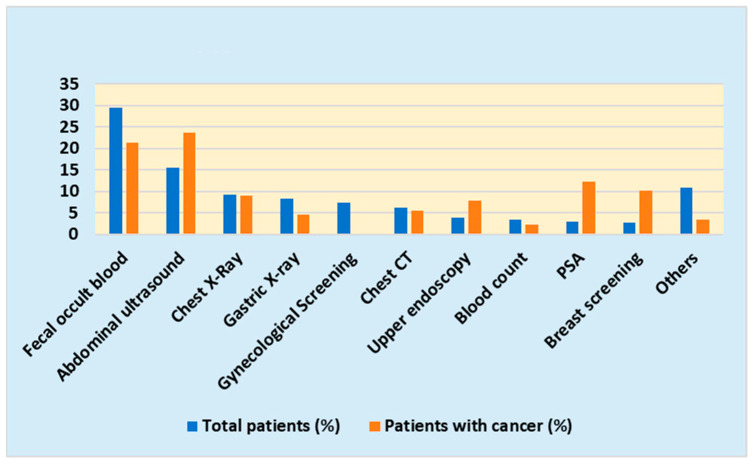
Reasons for hospital referral.

The reasons for hospital referrals (total number of patients and patients with cancer) are shown in Figure 2.

The most common reasons for referral were abdominal ultrasonography, fecal occult blood evaluation, gastric examination, chest radiography, chest CT, and gynecological examination (80% of the total cancer detections). Abdominal ultrasonography, fecal occult blood evaluation, and PSA assessment contributed to a large number of cancer detection cases, with these three alone accounting for 57% of cancer discoveries as the reason for referral.

In terms of correspondence to cancer type, stomach cancer was found on gastric ultrasonography, lung cancer on chest ultrasonography, and prostate cancer on PSA assessment, whereas a wide variety of cancers were found in 21 patients on abdominal echocardiography (6 patients with renal cancer, 4 with pancreatic cancer, 4 with bladder cancer, 3 with malignant lymphoma, 2 with prostate cancer, and 2 with gastrointestinal cancer).

**Figure 3:**
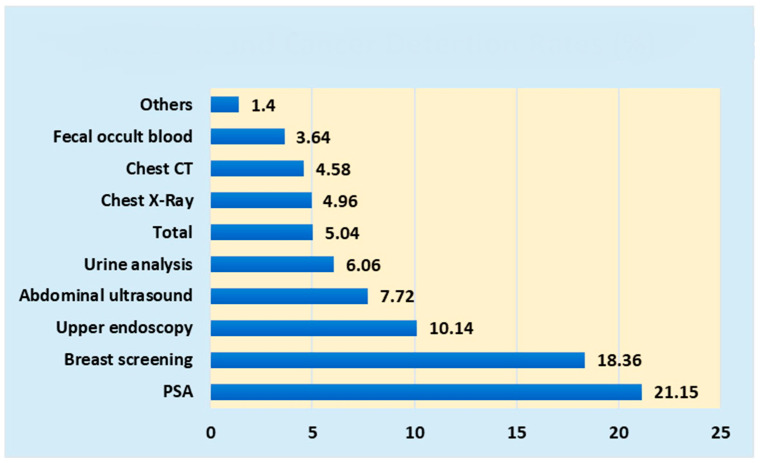
Reasons and cancer detection rates.

The reasons and cancer detection rates are shown in Figure 3.

The cancer detection rates were 21.15% for PSA assessment, 18.36% for breast screening, 10.14% for gastric endoscopy, and 7.72% for abdominal ultrasonography, with an overall average rate of 5.04%.

### 3.5. Identified Patients after the Survey

The number of patients (group before the survey) whose cancer was originally known by report or personal report was 33 (26 by report, 5 by personal report, and 2 unknown).

For the 56 referred patients (group after the survey) whose cancer was found after the medical record survey, the causes could not be identified before the survey.

(1) Results were not reported.
  Three (5.4%);
(2) An inspection was not performed when the report was prepared because of the time required to complete the inspection.
  Twenty-seven (48.2%);
(3) If the type of cancer (malignant or benign) was unclear, cancer was found after follow-up.
  Seven (12.5%);
(4) Referred to another hospital and found to have cancer.
  Eleven (19.6%);
(5) The initial biopsy was found to be benign but later proved to be malignant.
  Six (10.7%);
(6) Others
  Two (3.6%).

In addition, there were three cases in which cancer was discovered by chance as a result of referral to the Hospital for other causes, although they were not included in this year’s 89 patients.

(1) Referred for gynecological examination and surgery for benign tumors, during which a CT scan revealed lung cancer.
(2) Thorough examination of the lungs, during which CT revealed thyroid cancer.
(3) Referred for a thorough examination of the lungs, during which a PET-CT scan revealed liver cancer.

### 3.6. Effects of the Coronavirus Disease 2019 (COVID-19) Pandemic

**Figure 4:**
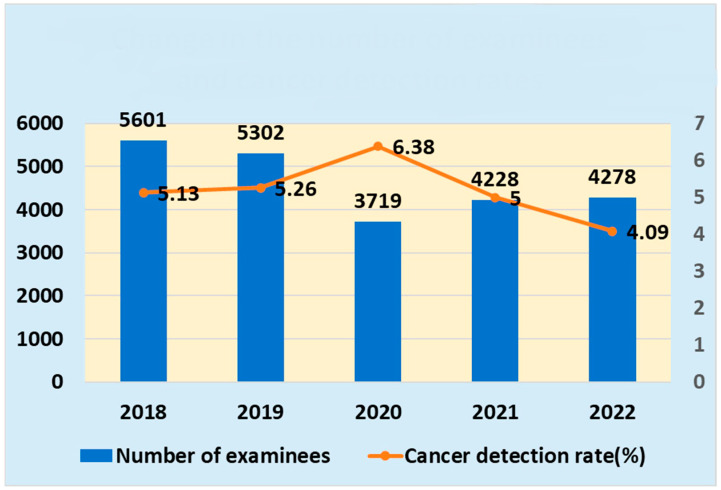
Change in the number of examinees and cancer detection rates.

The changes in the number of examinees and cancer detection rates are shown in Figure 4. 

The Center’s comprehensive health checkup showed an average of 493.3 examinees per month before the COVID-19 pandemic, but this number decreased to 363.5 cases per month after the pandemic (a 17.3% decrease). Similarly, the number of patients requiring medical examination decreased from an average of 59.0 per month before the pandemic to 48.0 after the pandemic (an 18.6% decrease). However, there was no decrease in the number of patients referred to the hospital, which averaged 30.1 per month before the pandemic and 30.6 per month after the pandemic. In other words, the rate of hospital referrals has increased among patients requiring medical examinations.

One-way analysis of variance showed no significant differences in cancer detection rates by year over the 5-year period from 2018 to 2022.

The COVID-19 pandemic reduced the Center’s comprehensive health checkup employees, but there was no difference in the cancer detection rate.

## 4. Discussion

In Japan, cancer screening is primarily conducted via public screening, which is promoted by the national government as a policy. This is because the Ministry of Health, Labour and Welfare (MHLW) has established the “Guidelines for Cancer Prevention and Health Education and Cancer Screening Implementation” (31 March 2008) and promotes cancer screening based on scientific evidence by municipalities [4]. Municipalities conduct cancer screening based on the Health Promotion Law, and public funds cover many of the costs. There are five types of cancer screening: gastric, cervical, lung, breast, and colorectal. The test items, participants, and consultation intervals were defined accordingly. The purpose of cancer screening is not only to detect cancer but also to reduce the mortality rate of the individuals (population) to be screened.

Five cancers (stomach, lung, breast, cervical, and colorectal) can be detected early by screening performed in a specific manner for each of these cancers, although there is some disagreement that further treatment reduces the mortality rate [8,9]. Therefore, these five types of cancer screening are official in Japan. However, in FY2020, the uptake rates for cancer screening conducted by municipalities were 7.0% for stomach cancer, 5.5% for lung cancer, 6.5% for colorectal cancer, 15.2% for cervical cancer, and 15.6% for breast cancer, which are low values [10].

In addition to these public cancer screenings, individual cancer screenings, such as comprehensive health checkups, are available in Japan. The contents vary from facility to facility, but it is hoped that performing various tests simultaneously will lead to early detection of various types of cancer [5].

According to the MHLW, in FY2020, the proportions of individuals diagnosed with cancer among those who were screened by the municipalities for cancer screening requiring close examination were 0.12% for stomach cancer, 0.03% for lung cancer, 0.17% for colorectal cancer, 0.02% for cervical cancer, 0.30% for breast cancer, 0.02% for uterine cervical cancer, and 0.30% for uterine cervical cancer. Breast cancer accounted for 0.30% [10].

The Japan Society of Ningen Dock, to which several comprehensive health checkup facilities belong, also published the number of cancers detected in patients undergoing comprehensive health checkups [6].

However, it is difficult to make uniform comparisons of cancer detection rates because of the differences in the conditions of each region and facility. The criteria for screening vary by country, region, facility, and screening type. In addition, the types of cancers covered widely vary during private cancer screenings.

According to Organization for Economic Co-operation and Development data, the cancer screening uptake rate for breast and cervical cancers in Japan is one of the lowest among developed countries; however, the mortality rate is low [11]. This can be due to the existence of systems, such as comprehensive health checkups other than public cancer screening, or to the high standard of medical care.

In this survey of 1763 patients referred from the Center to the Hospital over a 5-year period, the Center had identified 33 cancers prior to the survey, but the survey revealed an additional 56 cancers, resulting in a total of 89 cancers. The cancer detection rate increased from 1.87% before the survey to 5.05% after the medical record survey. The reasons for the identification of several cancers after the survey were also investigated.

Advanced cancers were often reported as cancers and responded to immediately after referral. However, several of the cancers that were discovered after the survey were written in the return form, such as “We will examine this more closely in the future.” In some cases, the test took longer, whereas in others, the initial biopsy was negative and later found to be positive. In some cases, such as at sites that were difficult to biopsy, cancer was identified after a considerable amount of time had passed.

In fact, the average time to confirm cancer diagnosis was ≤2.8 ± 2.5 months for those found before the survey but significantly longer for those found after the survey (≤4.8 ± 4.9 months). Regarding time to confirmed cancer diagnosis, the mean times to diagnosis were 4.98 ± 5.12 months for early-stage cancer and 2.28 ± 1.67 months for advanced cancer, indicating that early-stage cancer took significantly longer time to diagnosis than advanced cancer. This was thought to be because advanced cancers are often identified in the results report, whereas early-stage cancers are often unknown as malignant or benign and are identified after follow-up or close examination, such as biopsy. Thus, comprehensive health checkups and cancer screening may detect more early-stage cancers than actual cancer statistics reports because early-stage cancers take longer to diagnose. We believe that the same may happen in cases referred to as requiring further examination in public or private cancer screenings at other facilities. In other words, it is possible that more cancers than the cancer detection rates published by the Japanese MHLW and Japan Society of Ningen Dock are actually detected after screening.

Early-stage cancers require a longer time to be diagnosed than advanced cancers. In some cases, when the malignant–benign nature was unclear, careful follow-up led to the detection of early-stage cancer. In other words, we confirmed that proper follow-up after receiving a report from the referral hospital is extremely important for improving the cancer diagnosis rate of comprehensive medical checkups.

### 4.1. Differences between Public Cancer Screening and Comprehensive Health Checkups

The most common reasons for referral to our hospital were abdominal ultrasonography, occult blood in stool assessment, gastrointestinal examination, chest radiography, chest CT, and gynecological examination, accounting for 80% of the total cancer detections. Abdominal ultrasonography, fecal occult blood assessment, and PSA evaluation contributed to a large number of cancer detections after the examination, with these three alone accounting for 57% of all cancer detections. The detection rates were as follows: PSA evaluation, 21.15%; breast examination, 18.36%; gastrointestinal endoscopy, 10.14%; and abdominal ultrasonography, 7.72%.

In Japan, official screening for prostate cancer is available only in a few municipalities. However, PSA-based prostate cancer screening projects have suggested that PSA may be useful for the detection of early-stage prostate cancer [12]. In Korea, gastric endoscopy has the potential to detect gastric cancer early and prolong survival in patients aged < 40 years [13]. The effectiveness of abdominal ultrasonography screening for pancreatic cancer has also been suggested [14].

Abdominal ultrasonography, chest CT, PSA evaluation, and other tests are not performed during official cancer screening in Japan. A comprehensive health checkup, which allows these tests to be selected simultaneously, was estimated to significantly contribute to cancer detection. Abdominal ultrasonography, in particular, has once again become a major tool in cancer detection, with a wide variety of cancers being detected.

Public cancer screening covers five types of cancer: lung, stomach, colon, breast, and cervical cancers; however, referrals triggered by comprehensive health checkups are discovering several types of cancer. The ability to manage a wide variety of cancers is also considered an advantage of comprehensive health checkups.

According to a poll conducted by the Public Relations Office of the Cabinet Office, the reasons for the low cancer screening uptake rate in Japan include: people do not have time to undergo screening; people are confident about their health status and do not feel the need to undergo screening; and people can visit a medical institution whenever they are concerned [3,15]. It would be good if public cancer screening could be complemented by various types of private comprehensive health checkups [5].

Research should also be conducted on testing methods that contribute to the detection of early-stage cancer [12,13,14]. Although this study showed the usefulness of abdominal ultrasonography, we believe that it is more suitable for private health checkups, such as comprehensive health checkups, than group health checkups in terms of time and cost.

### 4.2. Effects of the COVID-19 Pandemic

During the COVID-19 pandemic, several healthcare providers had limited health checkups and nonemergency medical services to reduce the risk of spreading the infection. Cancer screening is also affected worldwide as individuals tend to avoid medical facilities.

Estimated breast, colorectal, and prostate cancer screening rates in the USA declined the most in April 2020 (breast, –90.8%; colorectal, –79.3%; prostate, –63.4%) and recovered by July [16]. Data from Ontario, Canada, showed a 41% decrease in breast, cervical, colorectal, and lung cancer screening programs in 2020 compared with 2019 [17]. A survey conducted by the Japan Cancer Society showed that Cancer Screening, an official tissue-based screening method, decreased by 27.4% in 2020 compared with 2019, recovered in 2021, and decreased by 10.3% in 2019 [18].

The Center’s comprehensive health checkups were also suspended for 2 months in April and May 2020, when the Japanese government declared a state of emergency [7]. After reopening, measures were taken to prevent infection and limit the number of examinees.

Prior to the COVID-19 pandemic, the average number of patients per month was 493.3 but decreased to 363.5 after the pandemic (a 17.3% decrease). Similarly, the average number of referrals requiring medical examination was 59.0 per month before the pandemic but decreased to 48.0 per month after the pandemic (an 18.6% decrease). The reason for the almost identical rate of decrease is thought to be that the content of the comprehensive health checkup itself has not changed; therefore, the proportion of those who need to be examined has also not changed.

However, there was no decrease in referrals to the Hospital, which averaged 30.1 per month before and 30.6 after the pandemic. In other words, the referral rate to the Hospital increased among those who required examination. The reasons for this may include an increase in the proportion of local residents among patients due to the COVID-19 pandemic and the fact that referrals to the nearest hospital were the easiest to make due to restrictions on medical care at hospitals in various regions.

One-way analysis of variance for cancer detection rates among the Hospital recipients showed no significant differences over the 5-year period from 2018 to 2022. The effects of COVID-19 on cancer screening detection rates may vary by region and time.

At the Center, although the number of examinees decreased, there was no decrease in the number of patients referred to the Hospital, and the cancer detection rate did not significantly change.

The decline in the number of people screened due to the COVID-19 pandemic was seen in cancer screening in Japan and around the world as well. Lessons learned from the COVID-19 pandemic continue to be sought in the state of cancer screening [19,20,21].

### 4.3. Study Limitations

This study had no information on lifestyle habits, especially smoking, exercise, and salt intake. This may have resulted in some effects of the multiple regression models shown in Table 3 and Table 6.

In addition, adjustments to the statistical analysis may not be sufficient.

## 5. Conclusions

Based on our results, the medical record survey demonstrated that cancer had been detected in more than twice as many individuals as were known from reports and other sources. Early-stage cancers require a longer time for diagnosis than advanced cancers. Therefore, careful follow-up leads to the detection of early-stage cancer. Proper follow-up after referral for a comprehensive health checkup is important. If these situations are similar to other cancer screenings, it is inferred that comprehensive health checkups and cancer screening contribute more to cancer detection than the statistical figures suggest. Abdominal ultrasonography, fecal occult blood evaluation, PSA assessment, and other tests are important triggers for cancer detection. Although public cancer screening provides only limited tests, physical examinations, which offer several tests to choose from, contribute to cancer detection. Although the number of examinees decreased before and after the COVID-19 pandemic, there is no difference in the cancer detection rate. In conclusion, short-term reports from the referring hospital are insufficient for a final diagnosis, and long-term follow-up is extremely important to increase the diagnosis rates of cancer for a comprehensive health checkup.

## Figures and Tables

**Table 1:** Basic information about 1763 patients referred to the Hospital.

Total (*n* = 1763)	Age (y) 62.0 ± 11.7		
	Men (*n* = 938)	Women (*n* = 825)	
Age (y)	64.3 ± 11.5	59.4 ± 11.4	*p*-value < 0.001 *
	Patients with cancer (*n* = 89)	Patients without cancer (*n* = 1674)	
No. of Before survey	33	1730	Cancer rate 1.87%
No. of After survey	33 + 56 = 89	1674	Cancer rate 5.05%

* *t*-test. Before, group before the survey; after, group after the survey. Cancer rate, cancer detection rate.

**Table 2:** Comparison of variables between patients with and without cancer.

	Patients with Cancer (*n* = 89)	Patients without Cancer (*n* = 1674)	*p*-Value
Age (y)	67.2 ± 8.8	61.7 ± 11.8	<0.001 *
Men/women	61/28	877/797	0.002 **
Before/after	33/89	1730/1674	<0.001 **

* *t*-test. ** Chi-squared test. Before, group before the survey; after, group after the survey.

**Table 3:** Relationship to the presence or absence of cancer.

	ß	OR	95% CI		*p*-Value	VIF
			Lower	Upper		
Year (For every one-year increment)	−0.081	0.922	0.798	1.065	0.269	1.009
Sex (Ref: Female)	0.486	1.626	1.017	2.599	0.042	1.046
Age (For every one-year increment)	0.042	1.043	1.020	1.065	<0.001	1.055
Constant	−4.702	0.009			<0.001	

Ref, reference; OR, odds ratio; VIF, variance inflation factor; Year, 2018–2022 year. Sex was considered a binary dummy variable (1/2), with males assigned a status of 1 and females assigned a status of 2.

**Table 4:** Comparison of variables between patients with cancer group before and after the survey.

	Before Survey (*n* = 33)	After Survey (*n* = 56)	*p*-Value
Age (y)	66.6 ± 9.2	67.7 ± 8.7	0.586 *
Time (within the month)	2.76 ± 2.45	4.80 ± 4.88	0.010 *
Early/advanced	17/13	33/12	0.134 **
Early/advanced + unknown	17/16	33/23	0.496 **

* *t*-test. ** Chi-squared test. Time, time to confirm cancer diagnosis; Early, early-stage cancer; Advanced, advanced cancer; Unknown, unknown stage cancer.

**Table 5:** Comparison of variables between patients with early-stage and advanced cancers.

	Early-Stage Cancer (*n* = 50)	Advanced Cancer (*n* = 25)	*p*-Value
Age (y)	66.5 ± 9.3	69.4 ± 6.4	0.090 *
Time (within the month)	4.98 ± 5.12	2.28 ± 1.61	0.001 *
Men/women	34/19	16/6	0.473 **

* *t*-test. ** Chi-squared test. Time, time to confirm cancer diagnosis.

**Table 6:** Stage and their correlation with risk factors between early-stage cancer and each factor.

	ß	OR	95% CI		*p*-Value	VIF
			Lower	Upper		
Survey (Ref: Before survey)	0.014	1.015	0.402	2.562	0.976	1.067
Year (For every one-year increment)	0.090	1.094	0.796	1.505	0.579	1.134
Age (For every one-year increment)	−0.027	0.973	0.922	1.026	0.317	1.101
Sex (Ref: Male)	−0.047	0.954	0.366	2.486	0.923	1.053
Time (within the month)	0.205	1.228	1.012	1.489	0.037	1.155
Constant	1.121	3.068	0.402	2.562	0.598	

Ref, Reference; OR, Odds Ratio; VIF, Variance Inflation Factor; Year, 2018–2022 year; Time, time to confirmed cancer diagnosis. Sex was considered a binary dummy variable (1/2), with males assigned a status of 1 and females assigned a status of 2.

## Data Availability

Data are contained within the article.

## References

[1] Ministry of Health, Labor, and Welfare National Health Insurance System. https://www.mhlw.go.jp/content/12400000/000377686.pdf.

[2] Ministry of Health, Labor, and Welfare Occupational Health and Safety Law Health Examination. https://www.mhlw.go.jp/file/05-Shingikai-11201000-Roudoukijunkyoku-Soumuka/0000136750.pdf.

[3] Ministry of Health, Labor, and Welfare Health Checkup. https://www.gov-online.go.jp/useful/article/201402/1.html#secondSection.

[4] Ministry of Health, Labor, and Welfare Cancer Screening. https://www.mhlw.go.jp/stf/seisakunitsuite/bunya/0000059490.html.

[5] Lu J. (2022). Ningen Dock: Japan’s unique comprehensive health checkup system for early detection of disease. Glob. Health Med..

[6] Committee to Survey Facilities Performing Comprehensive Health Checkups 2017/2018 Report on a Survey of Member Facilities (2021/3/31). https://www.ningen-dock.jp/wp/wp-content/uploads/2013/09/d36e0ceb105a39e3b9a9b519b10affd1-4.pdf.

[7] Yamanouchi Y., Maeda K., Shinoda Y., Majima M., Lee J., Inoue I., Maruyama Y., Kurabayashi H. (2022). Can outpatient rehabilitation be continued during the COVID-19 pandemic? A report from a Japanese regional medical university hospital. Arch. Rehabil. Res. Clin. Transl..

[8] Bretthauer M., Wieszczy P., Løberg M., Kaminski M.F., Werner T.F., Helsingen L.M., Mori Y., Holme Ø., Adami H.O., Kalager M. (2023). Estimated lifetime gained with cancer screening tests: A meta-analysis of randomized clinical trials. JAMA Intern. Med..

[9] Japan Cancer Society Promotion of Cancer Prevention and Cancer Screening. https://www.jcancer.jp/about_cancer_and_checkup.

[10] Ministry of Health, Labor, and Welfare Summary of FY2020 Community Health and Health Promotion Program Report. https://www.mhlw.go.jp/toukei/saikin/hw/c-hoken/20/dl/R02gaikyo.pdf.

[11] Cancer Care: Chart Set. https://web-archive.oecd.org/2014-02-06/265327-Cancer%20care%20(chart%20set).pdf.

[12] Uchida K., Akaza H. (1998). Characteristics of screening-detected prostate cancer on health checkup. Gan Kagaku Ryoho.

[13] Moon H.H., Kang H.W., Koh S.J., Kim J.W., Shin C.M. (2019). Clinicopathological characteristics of asymptomatic young patients with gastric cancer detected during a health checkup. Korean J. Gastroenterol..

[14] Yamaguchi A., Kato N., Sugata S., Hamada T., Furuya N., Mizumoto T., Tamaru Y., Kusunoki R., Kuwai T., Kouno H. (2022). Effectiveness of abdominal ultrasonography for improving the prognosis of pancreatic cancer during medical checkup: A single center retrospective analysis. Diagnostics.

[15] Government Public Relations Office, Cabinet Office Public Opinion Survey on Cancer Control. https://survey.gov-online.go.jp/h28/h28-gantaisaku/gairyaku.pdf.

[16] Chen R.C., Haynes K., Du S., Barron J., Katz A.J. (2021). Association of cancer screening deficit in the United States with the COVID-19 pandemic. JAMA Oncol..

[17] Walker M.J., Meggetto O., Gao J., Espino-Hernández G., Jembere N., Bravo C.A., Rey M., Aslam U., Sheppard A.J., Lofters A.K. (2021). Measuring the impact of the COVID-19 pandemic on organized cancer screening and diagnostic follow-up care in Ontario, Canada: A provincial, population-based study. Prev. Med..

[18] Japan Cancer Society Report No. 714. https://www.jcancer.jp/wp-content/uploads/TAIGAN-05-714_4c.pdf.

[19] Inoue D., Orisaka M., Hirose H., Miyashita H., Yamada S., Tsuyoshi H., Shinagawa A., Kurokawa T., Yoshida Y. (2023). Attitudes toward cancer screening in regional Japan during the COVID-19 pandemic: An anonymous survey. Cancer Sci..

[20] Barsouk A., Saginala K., Aluru J.S., Rawla P., Barsouk A. (2022). US Cancer Screening Recommendations: Developments and the Impact of COVID-19. Med. Sci..

[21] Maio F., Tari D.U., Granata V., Fusco R., Grassi R., Petrillo A., Pinto F. (2021). Breast Cancer Screening during COVID-19 Emergency: Patients and Department Management in a Local Experience. J. Pers. Med..

